# Polyphyllin Ⅲ-Induced Ferroptosis in MDA-MB-231 Triple-Negative Breast Cancer Cells can Be Protected Against by KLF4-Mediated Upregulation of xCT

**DOI:** 10.3389/fphar.2021.670224

**Published:** 2021-05-10

**Authors:** Yulu Zhou, Jingjing Yang, Cong Chen, Zhaoqing Li, Yongxia Chen, Xun Zhang, Linbo Wang, Jichun Zhou

**Affiliations:** ^1^ Department of Surgical Oncology, Sir Run Run Shaw Hospital, Zhejiang University School of Medicine, Hangzhou, China; ^2^ Biomedical Research Center and Key Laboratory of Biotherapy of Zhejiang Province, Hangzhou, China

**Keywords:** Polyphyllin III, ACSL4, ferroptosis, xCT, KLF4, breast cancer

## Abstract

Ferroptosis, which is characterized by the accumulation of intracellular iron and subsequent lipid peroxidation, is a newly discovered form of regulated cell death and plays an important role in tumor suppression. Herein, we showed that Polyphyllin III, which is a major saponin extracted from *Paris polyphylla* rhizomes, exerted its proliferation-inhibitory effect on MDA-MB-231 triple-negative breast cancer cells mainly through ACSL4-mediated lipid peroxidation elevation and ferroptosis induction. ACSL4 deletion partly attenuated Polyphyllin III-induced ferroptosis. Polyphyllin III treatment also induced KLF4-mediated protective upregulation of xCT, which is the negative regulator of ferroptosis. Interestingly, combination with the xCT inhibitor sulfasalazine (SAS) or downregulation of KLF4 sensitized MDA-MB-231 cells to Polyphyllin III. Furthermore, *in vivo* xenograft models, SAS significantly sensitized MDA-MB-231 breast cancer cells to Polyphyllin III, likely by enhancing intracellular lipid peroxidation and ferroptosis. The results of this study collectively demonstrated that Polyphyllin III exerts its anticancer effect by inducing ferroptosis via ACSL4 in MDA-MB-231 breast cancer cells. More importantly, we observed for the first time that KLF4-mediated xCT upregulation serves as negative feedback during ferroptosis progression, which might contribute to drug resistance in cancer treatment.

## Introduction

Breast cancer is one of the most common cancers among women worldwide, with an estimated 2.1 million new cases in 2018 (Bray et al., 2018). According to the National Cancer Institute, there were 276,480 estimated new cases and 42,170 estimated deaths of breast cancer in the United States in 2020 (National Institutes of Health, 2021). The latest report of cancer epidemiology in China also showed that breast cancer had an estimated annual incidence of 45.29/100 000 and a mortality rate of 10.50/100,000 (Zheng et al*.*, 2019).

Breast cancer is a highly heterogeneous tumor that requires individualized comprehensive treatment. Treatments of breast cancer include surgery, radiotherapy, endocrine therapy, chemotherapy, targeted therapy, etc. The choice of treatment and the prognosis of breast cancer are closely related to its pathological features. Breast cancer can be divided into four subtypes: luminal A, luminal B, HER-2 positive and triple-negative breast cancer (TNBC), as determined by an immunohistochemical (IHC) analysis of estrogen receptors (ER), progesterone receptors (PR), Ki-67 and epidermal growth factor receptor type 2 (HER-2) (Goldhirsch et al., 2011). The expressions of ER, PR and HER-2 are all negative in TNBC. TNBC is regarded as a more aggressive, more invasive, and harder-to-treat breast cancer, which in turn affects its survival rate and life expectancy. Since cancer cells lack necessary receptors, common treatments such as hormone therapy and HER-2 target therapy are ineffective (Waks and Winer, 2019). Using chemotherapy to treat triple-negative breast cancer is still an effective option.

Phytochemicals derived from natural products are also a promising source for developing new therapies for cancer due to their potential effectiveness and low toxicity. Studies have shown that many natural plant extracts can significantly inhibit the growth of breast cancer cells (Banik et al., 2017; Devassy et al., 2015; Kasiri et al., 2020; B.-M.; Kim et al., 2013). Polyphyllin III (PPIII), which is also known as dioscin, is a major saponin extracted from Paris polyphylla rhizomes. It has been shown to exert anti-proliferation effects on several kinds of cancers, including gastric cancer (Fang et al., 2015), hepatocellular cancer (Hu et al., 2016), lung cancer (He et al., 2014) and breast cancer (Wang et al., 2016; Li et al., 2017). Previous studies have proposed several mechanisms for its anticancer activities, including the induction of cell cycle arrest (Qian et al., 2018), apoptosis (Kim et al., 2014), autophagy (Wang et al., 2016), as well as GSDME-dependent pyroptosis (Ding et al., 2020). However, the exact molecular mechanism by which Polyphyllin III exerts its cytotoxicity in human breast cancer remains unclear.

Ferroptosis characterized by the accumulation of intracellular iron and subsequent lipid peroxidation is a newly discovered form of regulated cell death (Dixon et al., 2012). Ferroptosis does not present classic features of apoptotic or necrotic processes, such as caspase3 activation, chromatin fragmentation or nuclear pyknosis, and cell shrinkage or rupture. Instead, the mitochondria shrink significantly, and the membrane density increases during the process of ferroptosis (Brigelius-Flohé and Kipp, 2009). Ferroptosis has been found to play an important role in the induction of tissue injury and protective effects toward neurodegenerative diseases (Chen et al., 2017; Xie et al., 2019). In addition, the activation of ferroptosis also exhibits remarkable anticancer activity (Yu et al., 2017).

Ferroptosis is executed by the overwhelming accumulation of lethal lipid ROS (Dixon et al., 2012). Once the antioxidant capacity of cells decreases, lipid ROS induce iron-dependent oxidative cell death, or ferroptosis. Ferroptosis is pivotally controlled by the System Xc-/glutathione (GSH)/glutathione peroxidase 4 (GPX4) axis (Conrad et al., 2015). System Xc-, which is composed of a light chain (xCT, transcribed by SLC7A11) and a heavy chain (4F2, transcribed by SLC3A2), is a cystine/glutamate transporter that pumps cystine in exchange for intracellular glutamate (Huang et al., 2005). Cystine is essential for the synthesis of GPX4, which is the GSH-dependent lipid hydroperoxidase that catalyzes the degradation of hydrogen peroxide and inhibits the production of lipid ROS (Maiorino et al., 2018). Notably, xCT is often abnormally overexpressed in certain cancer cells (Koppula et al., 2017), thus making genetic or pharmacological inhibition of xCT a target for tumor suppression by ferroptosis induction. Erastin, sorafenib, and sulfasalazine (SAS) have been identified as xCT inhibitors and exert anticancer activity via ferroptosis induction (Timmerman et al., 2014; Sato et al., 2018; Zhou et al., 2019).

In addition to the System Xc-/GSH/GPX4 axis, other metabolic pathways, such as lipid synthesis, also affect ferroptotic processes. Lipid hydroperoxides generated by polyunsaturated fatty acids (PUFAs) in cell membranes could induce oxidative damage to cells once the intracellular REDOX balance is disturbed (Yang et al., 2016). Acyl-CoA synthetase long-chain family member 4 (ACSL4) functions as the acylase of arachidonic acid (AA) and is responsible for the proliferation, invasion and migration of certain breast cancer cells. ACSL-dependent modulation of phospholipids, particularly that of AA, was recently shown to be a critical determinant of sensitivity to ferroptosis (Yuan et al., 2016).

Krüppel-like factor 4 (KLF4), as one of the most important transcription factors in eukaryotes, participates in the regulation of cell proliferation, differentiation, embryo development, etc. (Ghaleb and Yang, 2017). In addition, KLF4 plays an important regulatory role in the occurrence and development of various cancers, including breast cancer (Wei et al., 2006; Jia et al*.*, 2018a; Jia et al*.*, 2018b). However, its role in breast cancer is still unclear and controversial. Aberrant overexpression of KLF4 is found in many breast cancer cells, indicating its relationship with early formation and steam cell property maintenance in breast cancer (Yu et al., 2011). On the other hand, KLF4 also inhibits the invasion and metastasis of breast cancer by inhibiting the EMT process (Nagata et al., 2016).

In this study, we explored the anticancer effects of Polyphyllin III on MDA-MB-231 human breast cancer both *in vitro* and *in vivo* and investigated its underlying mechanism of action. Our results showed that Polyphyllin III exerted anticancer activities against MDA-MB-231 cells mainly via ferroptosis induction mediated by ACSL4. Furthermore, Polyphyllin III treatment induced the protective increase of xCT through KLF4 upregulation, which leads to the resistance of breast cancer cells to Polyphyllin III. The ferroptosis inducer SAS or the knockdown of KLF4 by siRNA enhances the proliferation inhibitory effect of Polyphyllin III on breast cancer by downregulating xCT and promoting ferroptosis. Notably, our study proposed the role of KLF4-mediated protective elevation of xCT in ferroptosis and anticancer drug resistance for the first time.

## Materials and Methods

### Cell Culture

All human breast cancer cell lines (MDA-MB-231, HS578T, MCF-7, T47D, HBL-100, BT549, MDA-MB-453, and NHFB) and breast epithelial cell line HBL-100 were purchased from the American Type Culture Collection (ATCC) and stored in liquid nitrogen. MDA-MB-231 and HS578 T cells were cultured in DMEM supplemented with 10% fetal bovine serum, 4.0 mM l-Glutamine, and 100IU/ml penicillin and 100IU/ml streptomycin. MCF-7 and T47D cells were cultured in RPMI-1640 medium supplemented with 10% fetal bovine serum, 4.0 mM l-Glutamine, and 100IU/ml penicillin and 100IU/ml streptomycin. All cells were cultured in a humidified incubator (37°C, 5% CO_2_). The cell culture media and supplements were purchased from Thermo Fisher Scientific.

### Reagents

The corresponding reagents erastin (#HY-15763), sulfasalazine (#HY-14655), Z-VAD-FMK (#HY-16658B), E-necrosulfonamide (#HY-100573), ferrostatin-1 (#HY-100579), deferoxamine (#HY-D0903), and ciclopirox (#HY-B0450) were purchased from MedChemExpress. Liproxstatin-1 (#S7699) was purchased from Selleck. 3-Methyladenine (#189490) was purchased from Sigma Aldrich.

### Cell Viability Assay

Cell viability was evaluated using a Cell Counting Kit-8 purchased from Ape Bio (K1018). Cells were seeded in 96-well plates. After treatment, the medium was replaced with 100 μL fresh medium containing 10 μL CCK8 reagent and incubated in a humidified incubator (37°C, 5% CO_2_) for 2 h. The OD value was measured at 450 nm using a Thermo Scientific Spectrophotometer (1510–00712).

### Cell Death Assay

Propidium iodide (PI, Invitrogen) was used as a fluorescent signal for cell death. Polyphyllin III with or without Fer-1 treated cells were transferred to SFM supplemented with 5 mg/ml PI for PI imaging. After a 30 min incubation, bright-field and PI images were acquired.

### Western Blot Analysis

Cells were harvested 24 h after treatment with Polyphyllin III with or without SAS and lysed with radioimmunoprecipitation assay (RIPA) buffer supplemented with 1× PMSF. Western blotting was performed according to the standard protocol with primary antibodies including GPX4 (1:1,000, abcam, ab125066), SLC7A11 (1:500, CST, 12691s), KLF4 (1:1,000, Novus, NBP1-83940), and ACSL4 (1:1,000, Santa Cruz, sc-271800) and the secondary antibody (1:5,000, Bioss, IWR-1-endo). Equal protein sample loading was monitored using an anti-β-actin antibody (1:2,000, Santa Cruz, sc-47778). Reactive bands were visualized with Amersham Image 600.

### RNA Extraction and qRT-PCR

The total RNA was extracted using TRIzol reagent (Invitrogen) according to the manufacturer’s instructions. cDNA was synthesized using SuperScript Ⅱ Reverse transcriptase. Quantitative real-time PCR was performed using SYBR GreenER qPCR SuperMix universal, and triplicate samples were run on a Stratagene MX3000P qPCR system according to the manufacturer’s protocol. The threshold cycle (Ct) values for each gene were normalized to those of β-actin, and the 2^−ΔΔCt^ method was used for quantitative analysis. The primers used were as follows:

Q-SLC7A11-F: TCA​TTG​GAG​CAG​GAA​TCT​TCA
Q-SLC7A11-R: TTC​AGC​ATA​AGA​CAA​AGC​TCC​A
Q-ACSL4-F: CAT​CCC​TGG​AGC​AGA​TAC​TCT
Q-ACSL4-R: TCA​CTT​AGG​ATT​TCC​CTG​GTC​C
Q-KLF4-F: CGG​ACA​TCA​ACG​ACG​TGA​G
Q-KLF4-R: GAC​GCC​TTC​AGC​ACG​AAC​T
Q-β-actin-F: CTG​GAA​CGG​TGA​AGG​TGA​CA
Q-β-actin-R: AAG​GGA​CTT​CCT​GTA​ACA​ATG​CA

### Plasmid, siRNA and Transfection

Adenoviral KLF4 (Ad-KLF4) was transfected into cells to overexpress KLF4. The LacZ plasmid vector was used as a nonspecific negative control. Proteins Knockdown was performed by duplex oligos siRNA transfection with Lipofectamine 3,000 (Invitrogen, no. 2189668) in OPTI-MEM (Gibco, no. 2185849) for 24 h. The corresponding siRNA sequences used were as follows:

siACSL4#1: 5′- CCU​CUU​AUU​UGC​UGU​GAA​ATT -3′ (sense);
5′- UUU​CAC​AGC​AAA​UAA​GAG​GTT -3′ (anti-sense);
siACSL4#2: 5′- GCU​GCA​AAU​GCC​AUG​AAA​UTT -3′ (sense);
5′- AUU​UCA​UGG​CAU​UUG​CAG​CTT -3′ (anti-sense);
siKLF4#1: 5′-GCAGCUUCACCUAUCCGAUdTdT-3′ (sense);
5′-dTdTCGUCGAAGUGGAUAGGCUA-3′ (anti-sense);
siKLF4#2: 5′-dTdTCGUCGAAGUGGAUAGGCUA-3′ (sense);
5′-dTdTCUGGUCCGUGAUGGCAUUU-3′ (anti-sense).
sixCT#1: 5′- UGG​AGU​UAU​GCA​GCU​AAU​U -3′ (sense);
5′- AAU​UAG​CUG​CAU​AAC​UCC​A -3′ (anti-sense);
sixCT#2: 5′- GAG​GUC​AUU​ACA​CAU​AUA​U -3′ (sense);
5′- AUA​UAU​GUG​UAA​UGA​CCU​C -3′ (anti-sense);

### Lipid ROS Assay

The relative lipid ROS level in cells was assessed using C11-BODIPY dye (Thermo Fisher Scientific, D3861). Cells were treated with 5 μM C11-BODIPY for 30 min, harvested and washed twice with PBS. The sediment was resuspended in 500 μL PBS. Oxidation of the polyunsaturated butadienyl portion of the dye results in a shift of the fluorescence emission peak from ∼590 to ∼510 nm, which was detected by flow cytometry (BD Bioscience). The relative lipid peroxidation level was calculated using the median of each peak.

### GSH Assay

The relative GSH concentration in the cell lysates was assessed using a total Glutathione Assay Kit (Beyotime, S0052) according to the manufacturer’s instructions. The total glutathione content can be calculated by measuring the OD value at 412 nm.

### Transmission Electron Microscope

Cells cultured in a 6-well plate were fixed with a solution containing 2.5% glutaraldehyde in PBS for 24 h. After being washed in PBS, the cells were treated with 0.1% Millipore-filtered cacodylate-buffered tannic acid, postfixed with 1% buffered osmium, and stained with 1% Millipore-filtered uranyl acetate. After dehydration and embedding, samples were incubated in a 60°C oven for 24 h. Digital images were obtained using a transmission electron microscope [Thermo Fisher (FEI)].

### Immunohistochemistry

Tissue sections from clinical specimens (collected from Sir Run Shaw Hospital) or the indicated mouse models were fixed in 10% buffered formalin and embedded in paraffin. For IHC staining, tissue slides were deparaffinized in xylene and rehydrated in alcohol. Endogenous peroxidase was blocked with 3% hydrogen peroxide. Antigen retrieval was performed in a microwave with 0.1 M sodium citrate buffer (pH 6.0). Sections were then incubated overnight at 4 °C with antibodies against KLF4 (1:1,000, Novus, NBP1-83940), SLC7A11 (1:500, Abcam, ab37185) and 4-HNE (1:500, Abcam, ab48506). Antibody binding was detected using an HRP-DAB kit. Sections were then counterstained with Mayer’s hematoxylin and mounted onto coverslips. Images were acquired using polarized-light microscopy (Nikon, Eclipse 80I).

### Immunoprecipitation

The cells were washed with PBS, and ice-cold lysis buffer (300 μL per 55 cm^2^ dish) was added. Adherent cells were scraped off the dish using cell scrapers, and the cell suspension was held at 4°C for 30 min. The suspension was centrifuged in a microcentrifuge at 4°C, and the supernatant was transferred into a clear tube. Then 5× loading buffer was added to 50 μL lysate to serve as the input. Next, 40 μL of bead slurry was added to another 250 μL lysate and incubated overnight at 4°C. The mixture was centrifuged in a microcentrifuge at 4°C, and 1.5× loading buffer was added to the sediment to serve as the IP. Western blotting was performed according to the standard protocol with primary antibodies including SLC7A11 (1:500, CST, 12691s) and KLF4 (1:1,000, Novus, NBP1-83940) and the secondary antibody (1:5,000, Bioss, IWR-1-endo). Equal protein sample loading was monitored using an anti-β-actin antibody (1:2,000, Santa Cruz, sc-47778). Reactive bands were visualized with an Amersham Image 600.

### Xenograft Studies

Animal studies were reviewed and approved by the Ethics Committee for animal studies of Sir Run Shaw Hospital affiliated to Zhejiang University. MDA-MB-231 xenografts were established in 5 week-old BALB/C nude mice (Shanghai SLAC Laboratory Animal Corporation) by inoculating 1 × 10^6^ cells mixed with Matrigel (BD Biosciences) at a 1:1 ratio into the abdominal mammary fat pad. When the tumor reached 50–100 mm^3^, the mice were assigned randomly into different treatment groups (DMSO, PPIII, SAS, and PPIII + SAS groups), and each group consisted of 5 mice. PPIII (5 mg/kg/day) and SAS (200 mg/kg/day) were dissolved in dimethyl sulfoxide (DMSO), diluted in PBS, and then intraperitoneally injected into mice at a dose of 10 ml/kg/d once a day. The tumor sizes were measured every three days, and the tumor volumes were calculated as follows: length × width^2^ × 0.5. After 21 days of treatment, all mice were euthanized, and the tumors were surgically removed. Portions of the tumors were immediately fixed in 10% buffered formalin for immunohistochemistry.

### Software and Statistical Analyses

Flow cytometry data were analyzed using Flow Jo (X.10.0.7r2). Statistical analysis was performed using GraphPad Prism 6. The values are presented as the mean ± S.D. Statistical significance was determined using Student’s t-test. The significance of the correlation was determined using Pearson correlation analysis. *p* ≤ 0.05 was denoted as statistically significant.

## Results

### Polyphyllin III Induces Ferroptosis in Breast Cancer Cells

**FIGURE 1:**
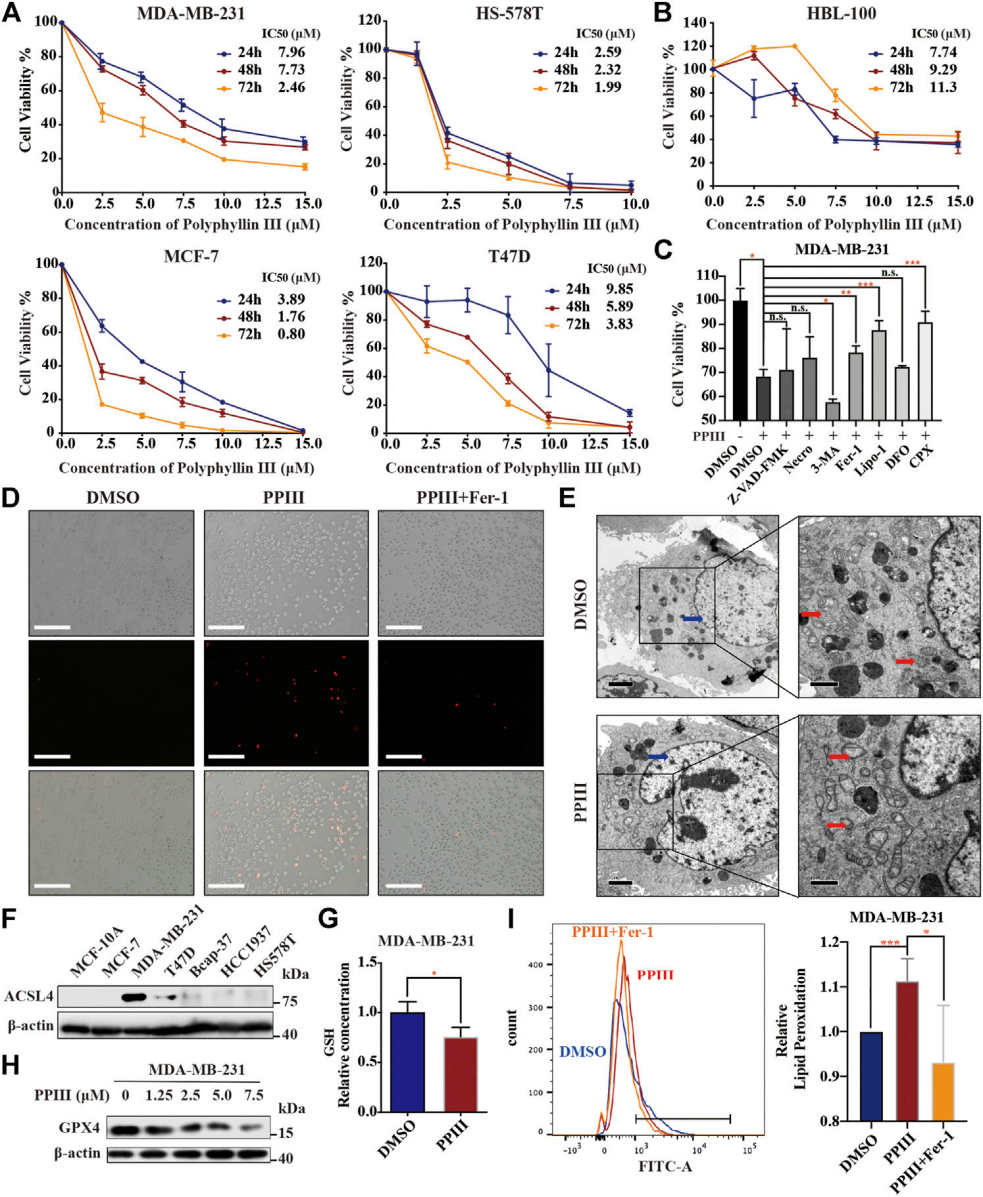
Polyphyllin III induces ferroptosis in breast cancer cells. **(A)**. MDA-MB-231, HS578T, MCF-7, and T47D cells were treated with different concentrations of Polyphyllin III for 24, 48 and 72 h (DMSO was added to replenish to the same volume). The cell viability was assayed (*n* = 3 independent repeats). **(B)**. HBL-100 breast epithelial cells were treated with different concentrations of Polyphyllin III for 24, 48 and 72 h (DMSO was added to replenish to the same volume). The cell viability was assayed (*n* = 3 independent repeats). **(C)**. MDA-MB-231 cells were treated with 5 μM Polyphyllin III for 24 h in the absence or presence of 20 μM Z-VAD-FMK, 1 μM (E)-necrosulfonamide (Necro), 10 μM 3-methyladenine (3-MA), 500 nM ferrostatin-1 (Fer-1), 200 nM liproxstatin-1 (Lipo-1), 50 μM deferoxamine (DFO), and 10 μM ciclopirox (CPX). The cell viability was assayed (*n* = 3 independent repeats, *p* values were calculated using two-tailed unpaired Student’s *t*-test). **(D)**. MDA-MB-231 cells were treated with 5 μM Polyphyllin III (PPIII) for 24 h in the absence or presence of 500 nM ferrostatin-1 (Fer-1). Propidium iodide (PI) staining for dead cells was conducted and observed under fluorescence microscopy. **(E)**. Transmission electron microscopy images of MDA-MB-231 cells treated with DMSO (Control) or 5 μM Polyphyllin III (PPIII) for 24 h. Red arrows, mitochondria; blue arrows, nucleus. **(F)**. The protein expression level of ACSL4 in different cell lines was analyzed by western blot. **(G)**. MDA-MB-231 cells were treated with DMSO or 5 μM Polyphyllin III (PPIII) for 24 h. The relative levels of GSH were assayed (error bars are means ± SD, *n* = 3 independent repeats, *p* values were calculated using a two-tailed unpaired Student’s t-test). **(H)**. Western blot analysis of GPX4 expression in MDA-MB-231 cells after Polyphyllin III (0–7.5 μM) treatment for 24 h. **(I)**. MDA-MB-231 cells were treated with 5 μM Polyphyllin III (PPIII) for 24 h in the absence or presence of 500 nM ferrostatin-1 (Fer-1). The relative levels of lipid peroxidation were assayed by C11-BODIPY fluorescence (the error bars show the means ± SD, *n* = 3 independent repeats, *p* values were calculated using a two-tailed unpaired Student’s t-test). *p < 0.05; **p < 0.01; ***p < 0.001; n. s., not significant (*p* > 0.05).

To detect the cytotoxicity of Polyphyllin III toward triple-negative breast cancer (TNBC), we treated MDA-MB-231 and HS-578 T human TNBC cells with various concentrations of Polyphyllin III (PPIII, from 2.5 to 15.0 μM) for 24, 48 and 72 h and determined the cell viability using CCK8 assays. Polyphyllin III exerted dose- and time-dependent toxicity and proliferation inhibitory effects on both cancer cell lines (Figure 1A). Polyphyllin III exerted similar dose and time-dependent toxicity on MCF-7 and T47D breast cancer cells (Figure 1A) but not HBL-100 normal breast epithelial cells (Figure 1B), indicating Polyphyllin III had exclusively an anticancer activity. Next, we explored which form of cell death dominated in Polyphyllin III-induced MDA-MB-231 cell death. During the expanding experiment, we observed that there were not enough cancer cells left for subsequent studies with 7.5 μM Polyphyllin III. Referring to relevant research (Wang et al., 2016; Ding et al., 2020), we combined 5 μM Polyphyllin III with various cell death inhibitors, including Z-VAD-FMK (an apoptosis inhibitor), (E)-necrosulfonamide (a necroptosis inhibitor), 3-methyladenine (3-MA, an autophagy inhibitor), ferrostatin-1 (Fer-1, a ferroptosis inhibitor), liproxstatin-1 (Lipo-1, a ferroptosis inhibitor), deferoxamine (DFO, an iron chelating agent), and CPX (ciclopirox, an iron chelating agent). The results showed that only the ferroptosis inhibitors Fer-1, Lipo-1, and CPX could significantly improve the cellular viability, while the others could not (Figure 1C). Moreover, it was observed via propidium iodide (PI) staining that Polyphyllin III-induced cell death could be inhibited by Fer-1 (Figure 1D), suggesting that Polyphyllin III might exert its cytotoxicity through ferroptosis in MDA-MB-231 breast cancer cells.

To clarify that Polyphyllin III induced ferroptosis, we first investigated the morphological changes in MDA-MB-231 cells. Transmission electron microscopy revealed that cancer cells after 24 h of Polyphyllin III treatment exhibited an increased density of the mitochondrial membrane and a decreased density of the mitochondrial cristae (red arrows), which are typical morphologic features of ferroptosis, compared to those after DMSO treatment (control). We did not observe cell shrinking or swelling after Polyphyllin III treatment, and no significant shrinkage of nuclei (blue arrows) or aggregation of chromatin was observed (Figure 1E). Other hallmarks of ferroptosis are the accumulation of intracellular lipid ROS and the decrease of GSH. Thus, we investigated the level of total glutathione, and the results showed a decrease in GSH levels after Polyphyllin III treatment (Figure 1G), followed by a decrease in GPX4 protein expression (Figure 1H). Lipid peroxidation assayed by C11-BODIPY fluorescence also showed that lipid peroxidation was increased after Polyphyllin III treatment, while the combination of Fer-1 could impede this phenomenon (Figure 1I).

### Polyphyllin III Induces Ferroptosis Partly by Upregulating ACSL4

**FIGURE 2:**
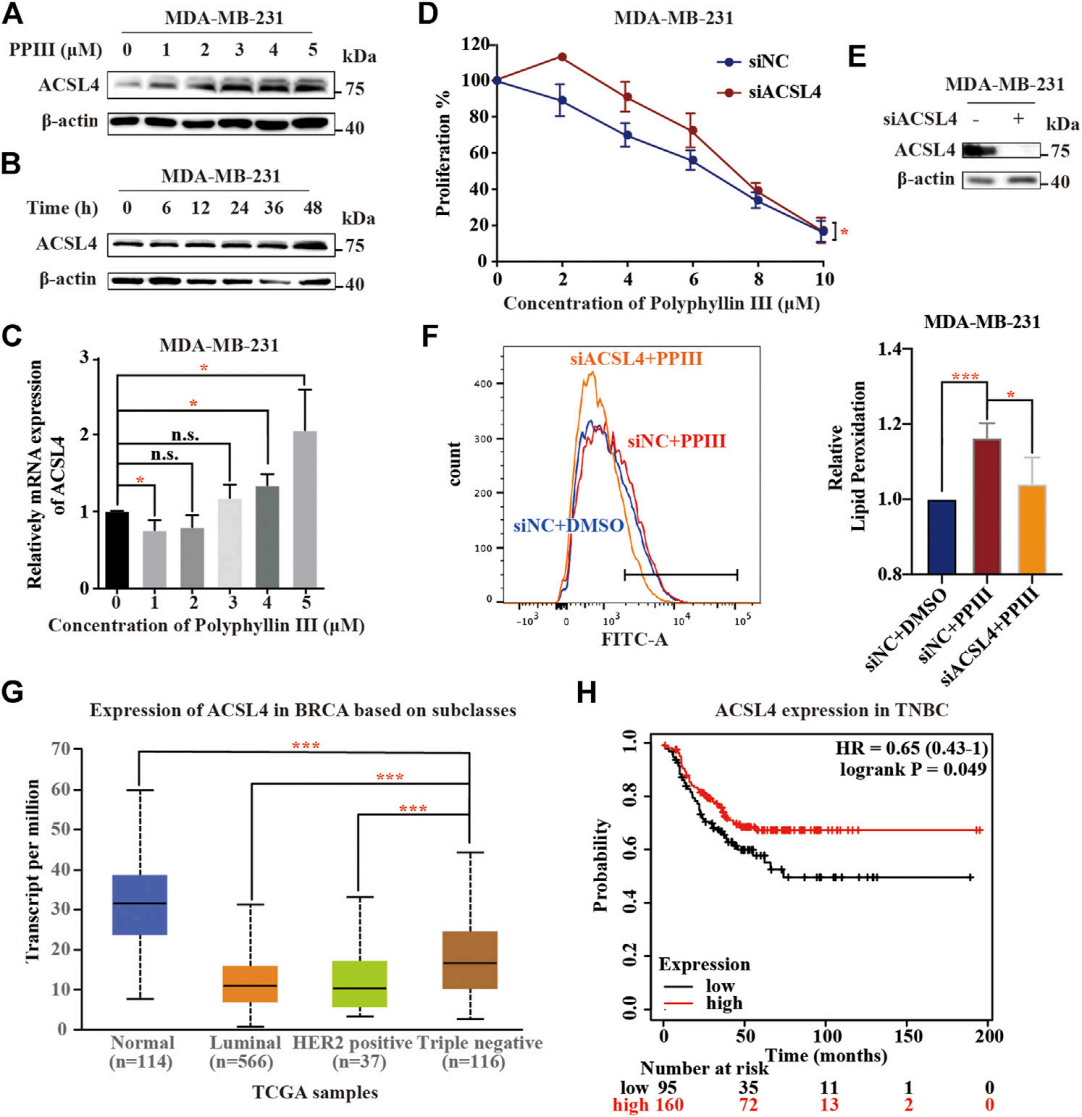
Polyphyllin III induces ferroptosis partly by upregulating ACSL4. **(A)**. Western blot analysis of ACSL4 expression in MDA-MB-231 cells after Polyphyllin III (0–5 μM) treatment for 24 h (DMSO was added to replenish to the same volume). **(B)**. Western blot analysis of ACSL4 expression in MDA-MB-231 cells after 5 μM Polyphyllin III treatment for 6, 12, 24, 36, and 48 h. **(C)**. The relative ACSL4 mRNA level was measured by qRT-PCR after MDA-MB-231 cells were treated with Polyphyllin III (0–5 μM) for 24 h (the error bars show the means ± SD, *n* = 3 independent repeats, *p* values were calculated using two-tailed unpaired Student’s t-test). **(D)**. MDA-MB-231 cells were transfected with negative control siRNA (siNC) or ACSL4 siRNA (siACSL4) and then treated with Polyphyllin III (0–25 μM) for 24 h. The cell viability was assayed (*n* = 3 independent repeats, *p* values were calculated using two-tailed paired Student’s t-test). **(E)**. Western blot analysis of ACSL4 expression in MDA-MB-231 cells after transfection with ACSL4 siRNA for 72 h. **(F)**. MDA-MB-231 cells were transfected with negative control siRNA (siNC) or ACSL4 siRNA (siACSL4) and then treated with 5 μM Polyphyllin III for 24 h. The relative levels of lipid peroxidation were assayed by C11-BODIPY fluorescence (the error bars show the means ± SD, *n* = 3 independent repeats, *p* values were calculated using a two-tailed unpaired Student’s t-test). **(G)**. Online UALCAN analysis of the expression of ACSL4 in BRCA based on subclasses. **(H)**. Online Kaplan-Meier Plotter analysis (http://kmplot.com/analysis) of triple-negative breast cancer patient outcomes. Differences in relapse-free survival (RFS) were compared in groups stratified by ACSL4 status. *p < 0.05; **p < 0.01; ***p < 0.001; n. s., not significant (*p* > 0.05).

Above, we revealed that Polyphyllin III induced ferroptosis in MDA-MB-231 cells. To demonstrate the associated mechanism, we first detected the expression of proteins related to the ferroptosis pathway and found that Acyl-CoA synthetase long-chain family member 4 (ACSL4) was upregulated after Polyphyllin III treatment, both dose- and time-dependently (Figures 2A,B). ACSL4 functions as an acylase of arachidonic acid (AA) to promote lipid peroxidation and plays an important role in ferroptosis. qRT-PCR also showed that ACSL4 was upregulated at the RNA level (Figure 2C). Next, we transfected MDA-MB-231 cells with small interfering RNA (siACSL4) to silence ACSL4 expression, as shown in Figure 2E. We showed that downregulation of ACSL4 attenuated Polyphyllin III-induced ferroptosis, as confirmed by cell viability assays and lipid ROS assays. ACSL4 silencing decreased the proliferation-inhibitory effect of Polyphyllin III on MDA-MB-231 cells (Figure 2D). Moreover, it partially reversed the Polyphyllin III-induced lipid peroxidation increase (Figure 2F). Together, our results indicated that Polyphyllin III induced ferroptosis partly through upregulating ACSL4. Online UALCAN analysis (http://Ualcan.path.uab.edu/analysis) of ACSL4 expression in BRCA based on subclasses showed that ACSL4 expression in BRCA cells was significantly lower than that in normal breast cells, while TNBC exhibited the highest ACSL4 expression level among all BRCA subclasses (Figure 2G), which matches our finding in Figure 2F. Online Kaplan-Meier Plotter analysis (http://kmplot.com/analysis) showed a correlation between the low expression of ACSL4 and a poor prognosis of patients with TNBC (Figure 2H), possibly because cancer cells were less sensitive to ferroptosis. These online analyses highlighted the future perspective of using Polyphyllin III in the treatment of breast cancer.

### Polyphyllin III Induces Protective xCT Upregulation, and Its Combination With SAS Sensitizes Breast Cancer Cells to Polyphyllin III

**FIGURE 3:**
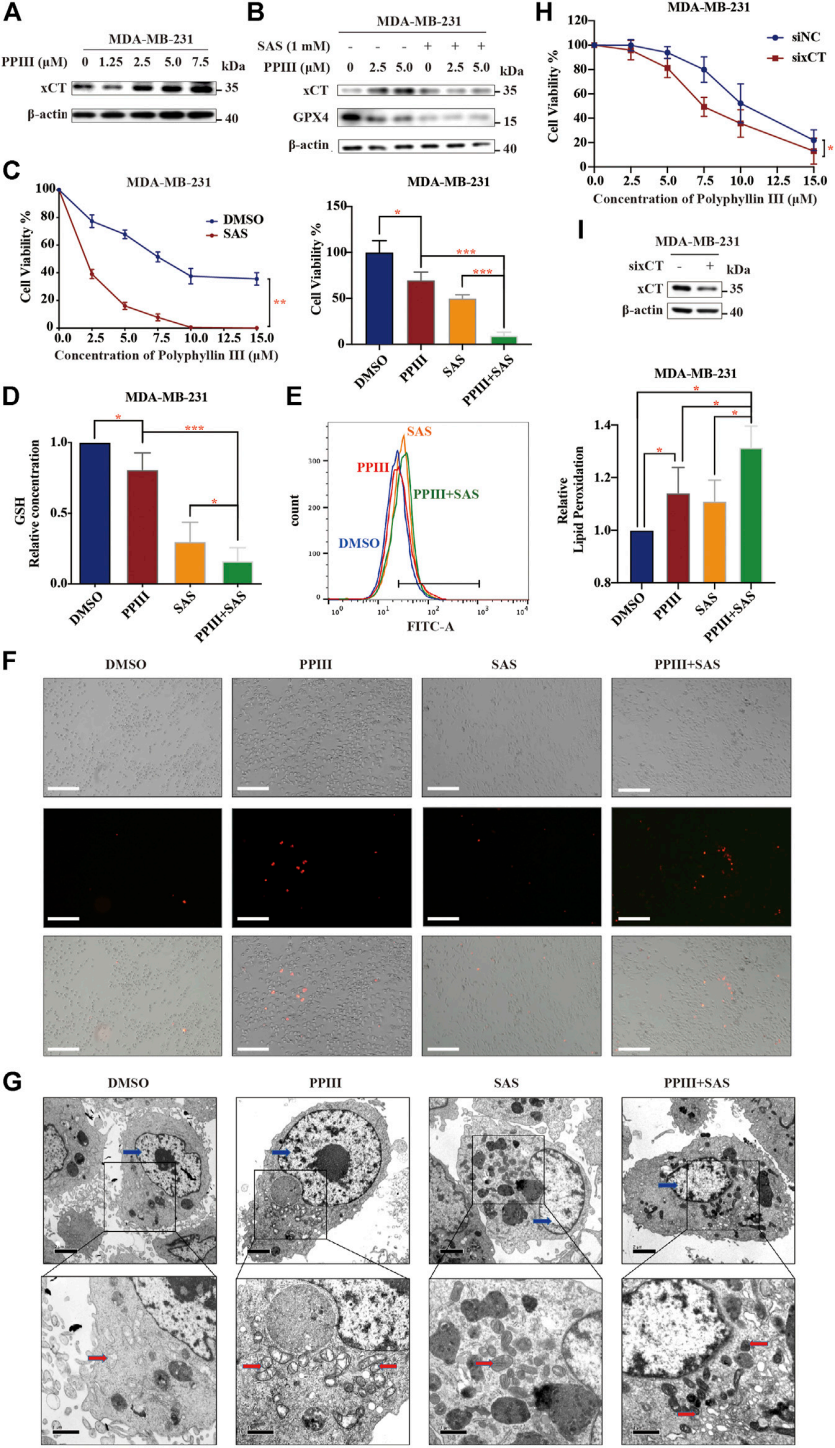
Polyphyllin III induces protective xCT upregulation, and its combination with SAS sensitizes breast cancer cells to Polyphyllin III. **(A)**. Western blot analysis of xCT expression in MDA-MB-231 cells after Polyphyllin III (0–7.5 μM) treatment for 24 h. **(B)**. Western blot analysis of xCT and GPX4 expression in MDA-MB-231 cells after Polyphyllin III treatment for 24 h with or without 1 mM SAS. **(C–G)**. MDA-MB-231 cells were treated with Polyphyllin III (0–15 μM) for 24 h in the absence or presence of 1 mM SAS. The error bars show the means ± SD, *n* = 3 independent repeats, and *p* values were calculated using a two-tailed unpaired Student’s t-test (C) The cell viability was assayed (D) The relative levels of GSH were assayed (E) The relative levels of lipid peroxidation were assayed by C11-BODIPY fluorescence (F) Propidium iodide (PI) staining for dead cells was conducted and observed under fluorescence microscopy (G) Transmission electron microscopy images were taken: red arrows, mitochondria; blue arrows, nucleus. **(H)**. MDA-MB-231 cells were transfected with negative control siRNA (siNC) or xCT siRNA (sixCT) and then treated with 5 μM Polyphyllin III for 24 h. The cell viability was assayed (*n* = 3 independent repeats, *p* values were calculated using two-tailed paired Student’s t-test). **(I)**. Western blot analysis of xCT expression after sixCT transfection. *p < 0.05; **p < 0.01; ***p < 0.001; n. s., not significant (*p* > 0.05).

When we detected the expression of ferroptosis-related proteins, we fortuitously observed the increasing expression of xCT after Polyphyllin III treatment (Figure 3A), which exerts an inhibitory effect on ferroptosis according to existing knowledge (Liu et al., 2020). We assumed that this protective upregulation of xCT could be an adaptive response of cancer cells to external stimulation, which might contribute to anticancer drug resistance.

We then combined Polyphyllin III with the xCT inhibitor sulfasalazine (SAS), an FDA-approved drug for ulcerative colitis, for the treatment of MDA-MB-231 cells. The results showed that after combination with SAS, the increase in xCT caused by Polyphyllin III treatment decreased (Figure 3B). More importantly, the sensitivity of cancer cells to Polyphyllin III was significantly increased, as confirmed by cell viability assays (Figure 3C) and PI staining (Figure 3F). In addition, SAS further increased the intensity of ferroptosis induced by Polyphyllin III with lower GSH levels and higher lipid peroxidation levels (Figures 3D,E). Morphologically, cancer cells treated with SAS and Polyphyllin III present more atrophic mitochondria, denser mitochondrial membranes and fewer mitochondrial cristae (Figure 3G) compared to those treated with a single application. We also used xCT siRNA (sixCT) to downregulate the expression of xCT (Figure 3I). Cell viability assay showed that sixCT strengthened the toxicity of Polyphyllin III on MDA-MB-231 cells (Figure 3H), further confirming the beneficial effect of xCT in Polyphyllin III activity, Together, our data strongly suggested that SAS sensitized MDA-MB-231 cells to Polyphyllin III, with a stronger intensity of ferroptosis.

### The Expressions of KLF4 and xCT Are Positively Correlated in Breast Cancer

Krüppel-like factor 4 (KLF4) is one of the most important transcription factors in eukaryotes and plays an important regulatory role in the occurrence and development of breast cancer. Studies have shown that changes in intracellular ROS could increase the expression of KLF4. We speculated that KLF4 might play a certain role in the induction of intracellular accumulation of lipid ROS and ferroptosis by Polyphyllin III.

**FIGURE 4:**
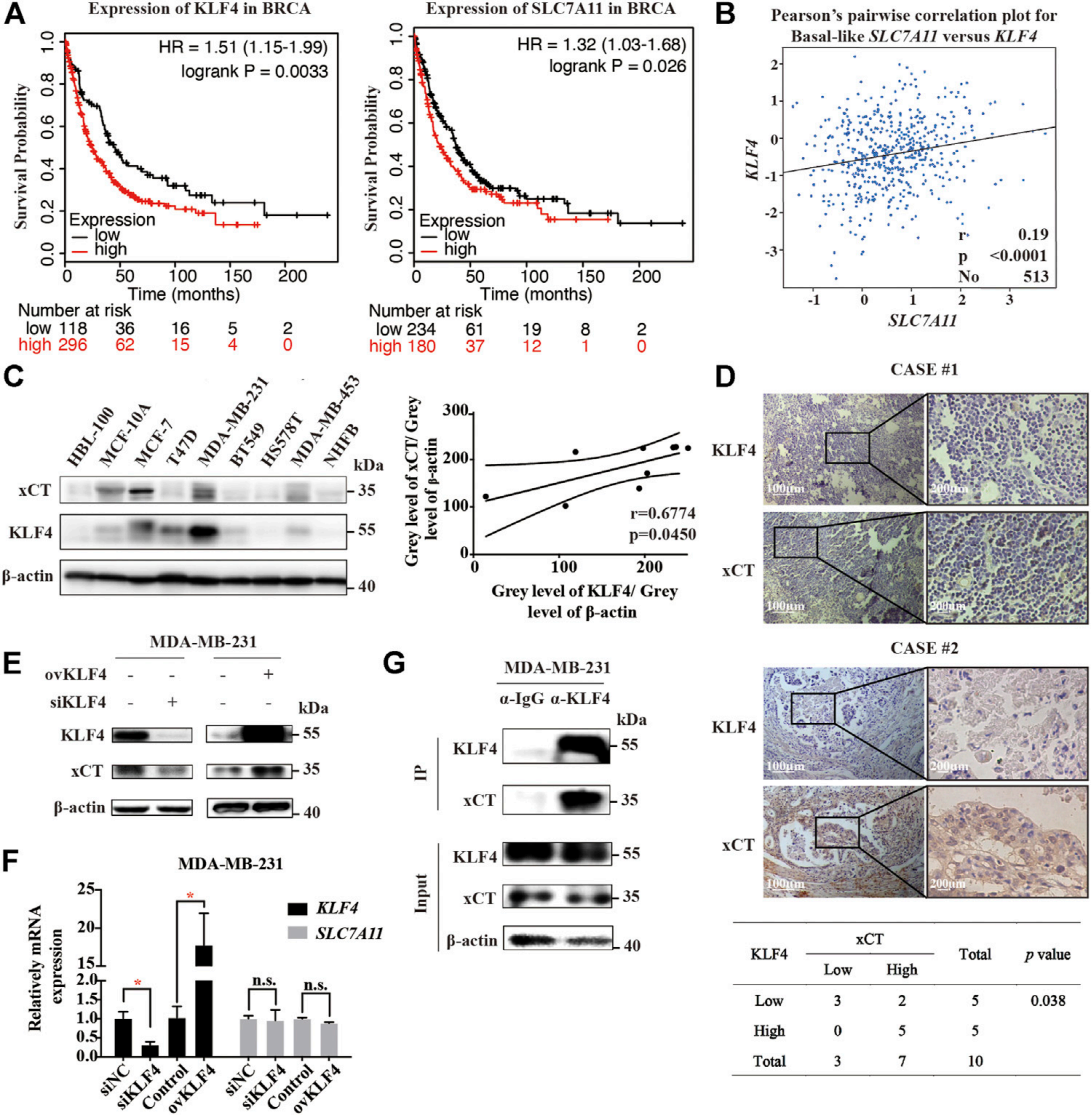
The expressions of KLF4 and xCT are positively correlated in breast cancer. **(A)**. Online Kaplan-Meier Plotter analysis (http://kmplot.com/analysis) of breast cancer patient outcomes. Differences in post progression survival (PPS) were compared in groups stratified by KLF4 or SLC7A11 status. **(B)**. Analysis of the GEO database shows that the expression levels of KLF4 and SLC7A11 in basal-like breast cancer cells are positively correlated. **(C)**. Western blot analysis of KLF4 and xCT expression in different breast cancer cell lines. The *p* value was calculated using Pearson correlation analysis. **(D)**. Immunohistochemical staining of KLF4 and xCT in clinical specimens of breast cancer patients. The correlation between KLF4 and xCT was assayed (*n* = 10, *p* value was calculated using Pearson correlation analysis). **(E)**. Western blot analysis of xCT expression after knocking down or overexpressing KLF4. **(F)**. qRT-PCR analysis of relative xCT mRNA expression after knocking down or overexpressing KLF4. **(G)**. The protein interaction between KLF4 and xCT was detected by immunoprecipitation. *p < 0.05; **p < 0.01; ***p < 0.001; n. s., not significant (*p* > 0.05).

First, we used online Kaplan-Meier Plotter analysis (http://kmplot.com/analysis) to analyse the relationship between breast cancer prognoses and mRNA expression levels of KLF4 or SLC7A11. In 414 breast cancer cases, both KLF4 and SLC7A11 mRNA levels were negatively related to patients’ post progression survival (PPS) (Figure 4A); that is, the higher the KLF4 or SLC7A11 mRNA expression level, the lower the survival rate after progression. Next, we performed data analysis on the GEO database. The results showed that KLF4 and SLC7A11 were positively correlated in basal-like breast cancer cells (Figure 4B). Then, KLF4 and xCT protein levels were measured in different breast cancer cell lines using western blotting analysis. We observed that the expression levels of both KLF4 and xCT in MCF-7, T47D and HS578T breast cancer cells were relatively high, while in BT549, MDA-MB-453 and NHFB breast cancer cells, they were relatively low (Figure 4C). Pearson correlation analysis showed that there was a significant positive correlation between KLF4 and xCT with a Pearson r value of 0.6774 and *p* < 0.05. Finally, 10 clinical specimens of breast cancer cases underwent immunohistochemical analysis of KLF4 and xCT. As shown in Figure 4D, the expression levels of KLF4 and xCT were also positively correlated, with the Pearson χ^2^ value of 4.286 and *p* < 0.05.

We then used small interfering RNA siKLF4 to silence or plasmid transfection to overexpress KLF4. Silencing KLF4 induced a decrease in xCT protein levels but not mRNA levels. Similarly, overexpressing KLF4 induced an increase in xCT protein levels but not mRNA levels (Figures 4E,F). Moreover, the protein interaction between KLF4 and xCT was detected by immunoprecipitation (IP), confirming that KLF4 had a direct interaction with xCT (Figure 4G).

### KLF4 Is Involved in Polyphyllin III-Induced Protective Upregulation of xCT

**FIGURE 5:**
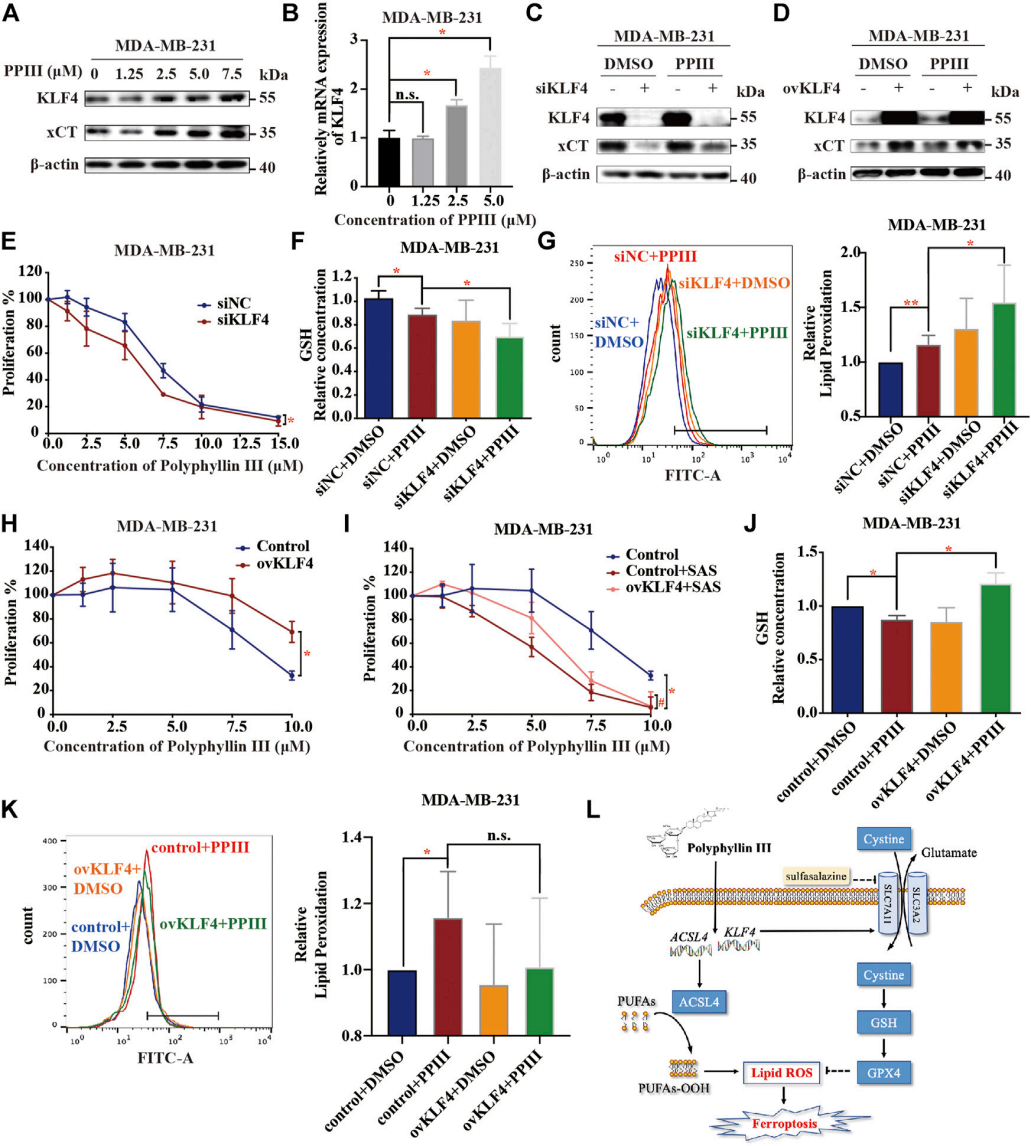
KLF4 is involved in Polyphyllin III-induced protective upregulation of xCT. **(A)**. Western blot analysis of KLF4 and xCT expression in MDA-MB-231 cells after Polyphyllin III (0–7.5 μM) treatment for 24 h. **(B)**. qRT-PCR analysis of relative KLF4 mRNA expression after Polyphyllin III (0–5 μM) treatment for 24 h. **(C,D)**. Western blot analysis of KLF4 and xCT expression after 5 μM Polyphyllin III treatment for 24 h when transfected (C) with negative control siRNA (siNC) or KLF4 siRNA (siKLF4) and (D) with the control plasmid (Control) or KLF4 plasmid (ovKLF4). **(E–G)**. MDA-MB-231 cells were transfected with negative control siRNA (siNC) or KLF4 siRNA (siKLF4) and then treated with 5 μM Polyphyllin III for 24 h. The error bars show the means ± SD, n = 3 independent repeats, and *p* values were calculated using a two-tailed paired Student’s t-test in (E) and a two-tailed unpaired Student’s t-test in (F) and (G) (E) The cell viability was assayed (F) The relative levels of GSH were assayed (G) The relative levels of lipid peroxidation were assayed by C11-BODIPY fluorescence. **(H–K)**. KLF4 was overexpressed using plasmid transfection in MDA-MB-231 cells, and then the cells were treated with 5 μM Polyphyllin III for 24 h. The error bars show the means ± SD, n = 3 independent repeats, *p* values were calculated using a two-tailed paired Student’s t-test in (H) and (I) and a two-tailed unpaired Student’s t-test in (J)–(K) (H) The cell viability was assayed (I) The cells were treated with 5 μM Polyphyllin III in the absence or presence of SAS for 24 h. The cell viability was assayed (J) The relative levels of GSH were assayed (K) The relative levels of lipid peroxidation were assayed by C11-BODIPY fluorescence. **(L)** Schematic diagram of the mechanism of Polyphyllin III in MDA-MB-231 cells. *p < 0.05; **p < 0.01; ***p < 0.001; n. s., not significant (*p* > 0.05).

Western blotting analysis confirmed that KLF4 was upregulated at both the protein (Figure 5A) and mRNA levels (Figure 5B). To demonstrate the hypothesis that KLF4 mediated Polyphyllin III-induced protective upregulation of xCT, transfection was conducted to silence or overexpress KLF4.

On one hand, silencing KLF4 decreased the xCT protein upregulation effect induced by Polyphyllin III (Figure 5C) and sensitized MDA-MB-231 cells to Polyphyllin III (Figure 5E). Silencing KLF4 further decreased the GSH levels (Figure 5F) and increased the lipid peroxidation levels (Figure 5G) compared to Polyphyllin III treatment only.

On the other hand, overexpressing KLF4 increased xCT protein expression (Figure 5D) and blocked the proliferation inhibitory effect caused by Polyphyllin III with (Figure 5H) or without SAS (Figure 5I). Overexpression of KLF4 compensated for the GSH reduction induced by Polyphyllin III (Figure 5J). Overexpression of KLF4 did decrease the lipid ROS elevation induced by Polyphyllin III (Figure 5K), but the difference was almost significance.

Together, our data showed that KLF4 was involved in Polyphyllin III-induced protective upregulation of xCT.

### SAS Contributed to the Tumor Suppression Effect of Polyphyllin III *in vivo*, Where Ferroptosis Was Involved

**FIGURE 6:**
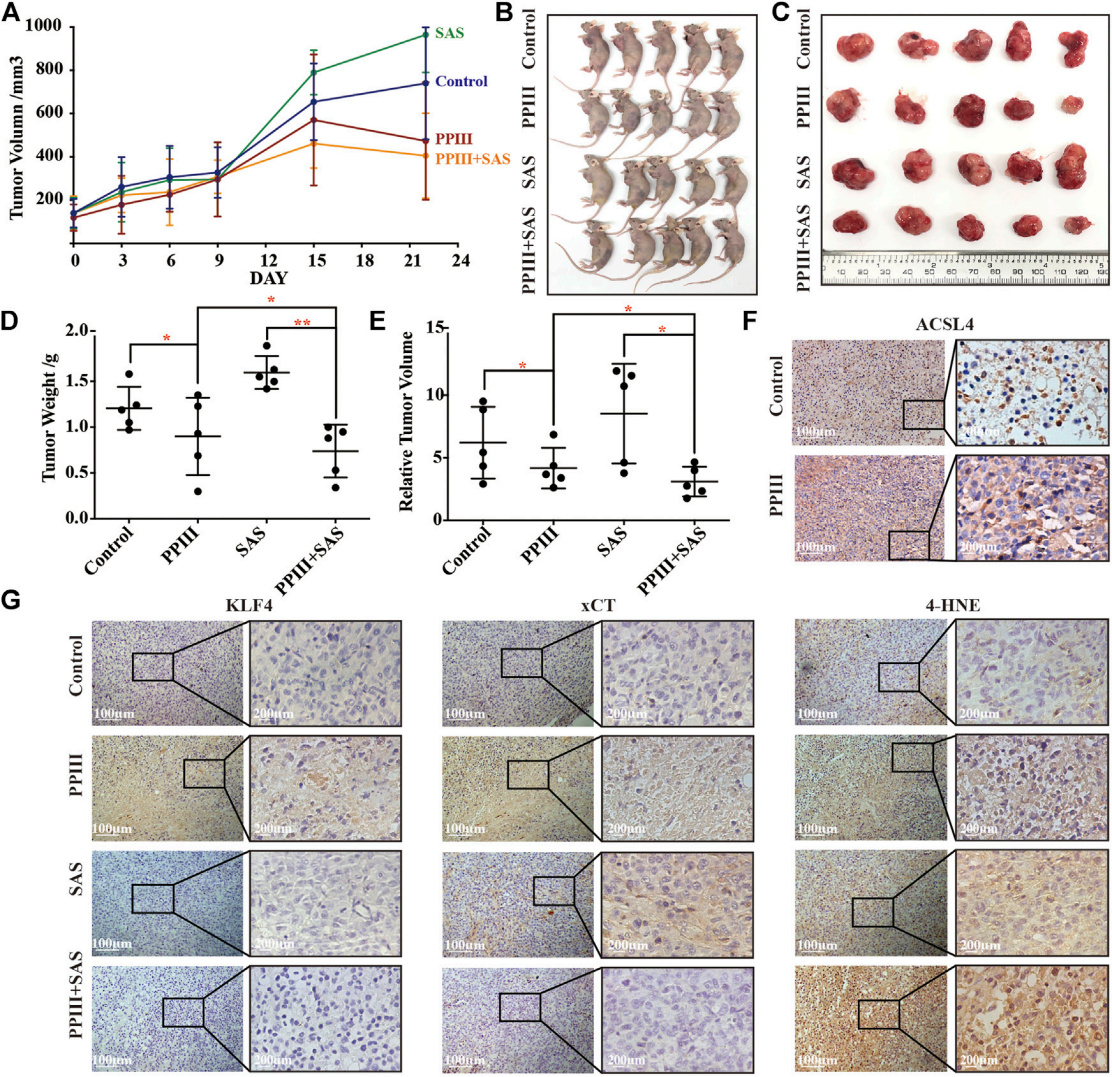
Polyphyllin III treatment with or without SAS suppressed tumor growth *in vivo*. **(A)**. Athymic nude mice were orthotopically injected with MDA-MB-231 cells and treated with Polyphyllin III and/or SAS daily by intraperitoneal injection until the end of experiments. The tumor volumes of MDA-MB-231 xenograft tumors at different time points (days) during the indicated treatments are shown. The error bars show the means ± SD, *n* = 5 independent repeats. *p* values were calculated using a two-tailed paired Student’s t-test. **(B)**. Images of the nude mice from each treatment group are shown at day 22 after the indicated treatment. **(C)**. Isolated tumor images from each treatment group are shown at day 22 after the indicated treatment. **(D)**. Individual value plot showing the weights of MDA-MB-231 tumor xenografts in each indicated treatment group at day 22. **(E)**. Individual value plot showing the relative tumor volumes of MDA-MB-231 tumor xenografts in each indicated treatment group at day 22. **(F)**. Immunohistochemical staining of ACSL4 in MDA-MB-231 xenograft tumors with or without Polyphyllin III treatment. **(G)**. Immunohistochemical staining of KLF4, xCT and 4-HNE in MDA-MB-231 xenograft tumors with the indicated treatments. Representative images of each group are shown. *p < 0.05; **p < 0.01; ***p < 0.001; n. s., not significant (*p* > 0.05).

Next, we explored the effect of Polyphyllin III with or without SAS *in vivo* by establishing a xenograft model of breast cancer in immunodeficient nude mice. MDA-MB-231 human breast cancer cells were implanted subcutaneously into the left axilla. Two weeks after implantation, the tumor-bearing mice were randomly divided into four groups: a negative control group (equal amount of DMSO dissolved in PBS), a PPIII group (5 mg/kg/day), an SAS group (200 mg/kg/day) and a PPIII + SAS group. Each group was treated daily with the indicated drugs intraperitoneally at a dosage of 200 ml/kg for 21 days. The tumor volume (Figures 6A,E) and tumor weight (Figure 6D) of the mice were recorded for assessment of the drug efficacy. As expected, Polyphyllin III potently suppressed tumor growth, and its combination with SAS further enhanced this effect on breast cancer (Figures 6B–E). Notably, SAS alone did not suppress the tumor growth of the xenografts. Immunohistochemical analysis of the tumor tissue confirmed the increase in ACSL4 expression after Polyphyllin III treatment (Figure 6F). Moreover, Polyphyllin III treatment significantly increased the expression of KLF4 and xCT, while the xCT inhibitor SAS combination reversed this phenomenon and enhanced the upregulation effect of Polyphyllin III on 4-HNE, which is an indicator of intracellular lipid peroxidation and oxidative stress (Figure 6G). Together, our data showed that Polyphyllin III exerted a tumor suppression effect *in vivo*. Moreover, the xCT inhibitor SAS significantly sensitized MDA-MB-231 breast cancer cells to Polyphyllin III, likely by enhancing intracellular lipid peroxidation and ferroptosis.

## Discussion

*Paris polyphylla*, which is also known as Chong-lou, is a traditional Chinese medical herb of the Liliaceae family. Chong-lou is prescribed to cure mastitis, sore throat, convulsion, tuberculous meningitis, and tumors of the respiratory system, digestive tract, liver, pancreas, urinary bladder, and brain, as well as breast cancer. Studies have revealed that many compounds extracted from Paris polyphylla can inhibit tumor proliferation (Lee et al., 2005; He et al., 2016; Li et al., 2017). As one of the main components of Paris polyphylla, the antitumor effect of Polyphyllin III on human breast cancer and its mechanism are not yet clear. In this study, we proposed that Polyphyllin III exerted a proliferation inhibitory effect on MDA-MB-231 triple-negative breast cancer cells mainly through ACSL4-mediated ferroptosis induction. ACSL4 deletion partly attenuated Polyphyllin III-induced ferroptosis. Moreover, Polyphyllin III treatment also induced protective upregulation of xCT, a negative regulator of ferroptosis, which in turn attenuated the intensity of Polyphyllin III-induced ferroptosis. Further studies discovered the correlation between KLF4 and xCT, in which KLF4 could directly interact with xCT and regulate its protein expression level. Polyphyllin III treatment of MDA-MB-231 cells increased KLF4 expression at the transcriptional level, which induced the upregulation of xCT and partial inhibition of ferroptosis. Combination with the xCT inhibitor sulfasalazine (SAS) or downregulation of KLF4 sensitized MDA-MB-231 cells to Polyphyllin III. Last, studies using *in vivo* xenograft models also presented similar results. Polyphyllin III potently suppressed tumor growth, and its combination with SAS further enhanced this effect. SAS significantly sensitized MDA-MB-231 breast cancer cells to Polyphyllin III, likely by enhancing intracellular lipid peroxidation and ferroptosis. Based on these results together, this study demonstrated that Polyphyllin III induced ferroptosis via ACSL4 in MDA-MB-231 breast cancer cells. More importantly, we observed for the first time that KLF4-mediated xCT upregulation served as negative feedback during ferroptosis progression (Figure 5L), which might contribute to drug resistance in cancer treatment.

However, several questions remain unsolved in our study. First, our study shows that Polyphyllin III induces ACSL4 expression, which is critical for PUFA metabolism and lipid peroxidation. The elevation of lipid ROS and the reduction of GPX4 also confirm the biological effect of ACSL4 upregulation. Furthermore, online UALCAN analysis and Kaplan-Meier Plotter analysis show a correlation between ACSL4 expression and breast cancer prognosis. However, the exact mechanism by which Polyphyllin III treatment influences ACSL4 is still unclear. Given the finding that both the protein and mRNA levels of ACSL4 increase, further studies could aim to identify transcriptional regulatory factors, transcription-associated enzymes, or DNA modifications, such as DNA methylation and histone modification. In addition, silencing ACSL4 expression only partly inhibits Polyphyllin III induced ferroptosis, indicating that there are additional pathways participating in the tumor suppression effect of Polyphyllin III on breast cancer cells.

Ferroptosis is pivotally controlled by the System Xc-/GSH/GPX4 axis (Conrad et al., 2015). xCT, which is the light chain composing System Xc-, is often abnormally overexpressed in certain cancer cells and is related to worse survival outcomes in breast cancer patients (Timmerman et al., 2014; Koppula et al., 2017). In our study, we identified the upregulation of xCT after treatment with the anticancer drug Polyphyllin III as a protective mechanism when cancer cells are under oxidative stress, such as in ferroptosis induction. A recent article published in Cell Research also pointed out that ionizing radiation (IR) induced an adaptive response involving SLC7A11 or GPX4 induction that promoted cancer cell survival (Lei et al., 2020). As a result, the addition of the xCT inhibitor SAS remarkably sensitizes cancer cells to Polyphyllin III or IR treatment, confirming the existence of such protective feedback. Other xCT inhibitors, including erastin and sorafenib, or GPX4 inhibitors, including RSL3 and FIN56, are also worth studying for their ability to inhibit ferroptosis. For example, Li et al. discovered that Nrf2/xCT pathway activation was associated with the resistance of cancer cells to cisplatin and demonstrated that erastin/sorafenib could induce ferroptosis in cisplatin-resistant non-small cell lung cancer cells, effectively enhancing the effect of chemotherapy (Li et al., 2020).

KLF4 is an important transcription factor in eukaryotes, participating in the regulation of cell proliferation, differentiation, embryo development, and the occurrence and development of tumors (Ghaleb and Yang, 2017). Our study found that KLF4 could upregulate xCT at the protein level but not at the mRNA level. Given the results of IP analysis indicating that KLF4 directly interacted with xCT, we preliminarily concluded that KLF4 regulated xCT protein expression at the posttranscriptional level. Therefore, how KLF4 specifically interacts with xCT and regulates its expression is worth further exploration. Meanwhile, the driving factor of KLF4 upregulation after Polyphyllin III treatment has not yet been fully understood. Sunaga et al. demonstrated that fatty acid metabolism could induce ROS production and AMPK/KLF4 signaling activation (Sunaga et al., 2016). Zhang et al. found that exogenous H2O2 could also significantly increase the levels of KLF4 in myocardial cells (Zhang et al., 2017). Considering our findings above, we made a reasonable speculation that intracellular lipid ROS induced by Polyphyllin III treatment not only activated ferroptosis but also increased KLF4. KLF4-mediated protective upregulation of xCT might be a critical negative feedback mechanism during the ferroptotic process that causes anticancer drug resistance. Of course, more research is needed to verify this hypothesis and to elucidate how cancer cells induce KLF4 upregulation in response to exogenous stimulation or endogenous oxidative stress. Understanding the mechanism by which cancer cells develop protective adaptation will provide new ideas for the development of anticancer drugs and strategies by which to address drug resistance.

## Data Availability

The raw data supporting the conclusions of this article will be made available by the authors, without undue reservation.
